# Tunable and high-sensitivity sensing based on Fano resonance with coupled plasmonic cavities

**DOI:** 10.1038/s41598-017-10626-1

**Published:** 2017-09-06

**Authors:** Yan Deng, Guangtao Cao, Hui Yang, Guanhai Li, Xiaoshuang Chen, Wei Lu

**Affiliations:** 1 College of Physics, Mechanical and Electrical Engineering, Jishou University, Jishou, 416000 China; 2 National Laboratory for Infrared Physics, Shanghai Institute of Technical Physics, Chinese Academy of Sciences, 500 Yu Tian Road, Shanghai, 200083 China; 3 University of Chinese Academy of Science, No. 19A Yuquan Road, Beijing, 100049 China

## Abstract

Tunable and high-sensitivity sensing based on Fano resonance is analytically and numerically investigated in coupled plasmonic cavities structure. To analyze and manipulate the Fano line shape, the coupled cavities are taken as a composite cavity that supports at least two resonance modes. A theoretical model is newly-established, and its results agree well with the finite difference time domain (FDTD) simulations for the plasmonic stub-pair structure. The detection sensitivity factor in coupled cavities approaches 6.541 × 10^7^ m^−1^, which is an order of magnitude larger than single stub case. In addition, the wavelengths of resonant modes in the plasmonic stub-pair structure can be adjusted independently, which paves a new way for improving detection sensitivity. These discoveries hold potential applications for realizing tunable and highly integrated photonic devices.

## Introduction

Fano resonance, originating from destructive interference between narrow discrete state and broad continuum state, exhibits sharp asymmetric profiles and strong field enhancement^1–3^, which may benefit enhanced spectroscopy^4^, optical switches^5^, chemical or biological sensing^6, 7^, low-loss waveguiding, and nonlinear optical effects. Surface plasmon polaritons (SPPs) can break the classical diffraction limit and enable manipulating light in sub-wavelength structures, so diverse plasmonic nanostructures such as core-shell nanoparticles^8^, ring-disk cavities^9^, nano-slits^10, 11^ and nanowire lattices^12^ have been proposed theoretically and experimentally to achieve Fano resonance. However, the arrayed structures are typically bulky for highly integrated optical circuits, which motivates an ongoing search for ultra-compact plasmonic structure realizing Fano resonance.

Among various plasmonic structures, metal-dielectric-metal (MDM) waveguide, supporting modes with deep subwavelength scale, ease of fabrication, and zero bend losses^13–16^, promises the miniaturization of optical devices and has attracted much attention. Recently, the optical properties of MDM waveguide-cavity systems exhibit symmetrical or asymmetrical line shapes, and have been studied by the scattering matrix method^17^, transmission line theory^18^, quantum-optics approach^19, 20^, and temporal coupled-mode theory (CMT)^17, 21^, which are considered as a crucial step for development of integrated photonic circuits^22–26^. It is well known that, with variation of nearby or surrounding medium, the transmission and reflection change at fixed wavelength or mode shift can be utilized as sensing signals. In particular, the sharp asymmetrical Fano resonance supported by coupled cavities enables high detection sensitivity in information processing^6, 7, 27–29^. Lu *et al*. demonstrated a nanosensor in MDM waveguide-cavity system^30^ and yielded a figure of merit (FOM) of ~500. Chen *et al*. proposed a near-infrared plasmonic refractive index sensor which consists of a fillet cavity coupled with two MDM waveguides^31^. In addition, Huang and co-workers introduced slow-light enhanced refractive index sensor composed of plasmonic MDM waveguide^32^. However, more feasible theoretical model and straightforward approach are desirable to control and tune the spectral response.

In this paper, we numerically and analytically demonstrate tunable and high-sensitivity sensing performances based on Fano resonance in plasmonic coupled cavities system. The coupled cavities are interpreted as a composite cavity, in which the interference between coexisting modes contributes to Fano line shape^20, 33^. A newly-established theoretical model is in good agreement with the FDTD simulations for the plasmonic stub-pair structure. Compared with single cavity case, the coupled plasmonic cavities structure exhibits much sharper asymmetric Fano line shape, and produces an order of magnitude enhancement in detection sensitivity. Moreover, the resonant positions of bright mode and dark mode in the stub-pair structure can be adjusted independently, which promises solutions for realizing and tuning the high detection sensitivity.

## Theoretical model

**Figure 1:**
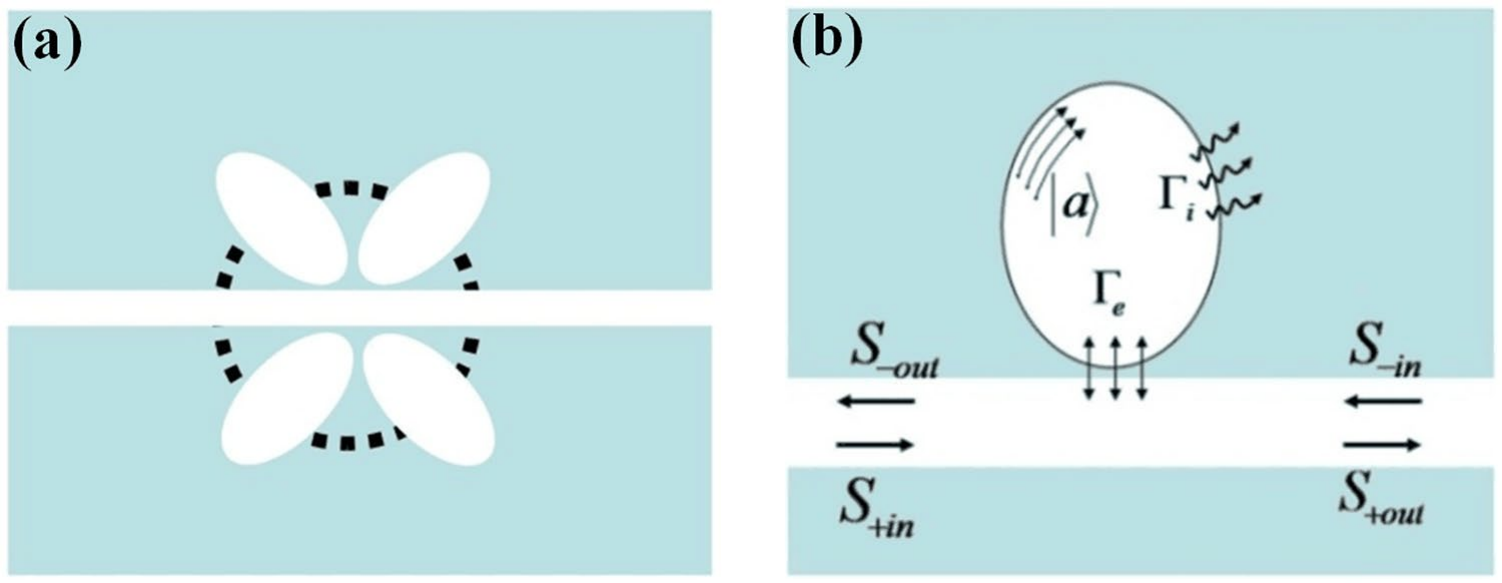
(**a**) Schematic of waveguide coupled to multiple cavities. (**b**) Schematic of theoretical model.

Figure 1(a) shows the schematic (sectional view) of coupled cavities structure, which is composed of a bus waveguide coupled to multiple resonators. The indirect couplings between resonators are not discussed here. In order to control and tune Fano resonance spectra in coupled cavities system, the coupled cavities are creatively treated as a composite cavity, as shown in Fig. 1(b). An analytical theory based on the CMT^17, 21^ is proposed to explore the underlying physics of spectral responses. Then, the characteristic equations for the evolutions of cavity modes can be expressed as follows

1 $$\frac{{\rm{\partial }}}{{\rm{\partial }}t}|a\rangle=-j{\rm{\Omega }}|a\rangle-{{\rm{\Gamma }}}_{i}|a\rangle-{{\rm{\Gamma }}}_{e}|a\rangle+{S}_{+in}|{\rm{K}}\rangle+{S}_{-in}|{\rm{K}}\rangle-{\rm{M}}|a\rangle,$$

2 $${S}_{+out}={S}_{+in}-\langle {\rm{{\rm K}}}|a\rangle ,$$

3 $${S}_{-out}={S}_{-in}-\langle {\rm{{\rm K}}}|a\rangle .$$

*S*_±*in*_ and *S*_±*out*_ stand for the amplitudes of incoming and outgoing waves in bus waveguide. $|a\rangle$ and $|{\rm{K}}\rangle$ represent field amplitude of resonant mode and coupling coefficient between resonator and bus waveguide, respectively $\langle {\rm{K}}|$ is dependent on $|{\rm{K}}\rangle$. They can be written as

$$|a\rangle =(\begin{array}{c}{a}_{1}\\ {a}_{2}\\ \vdots \\ {a}_{N}\end{array}),\quad |{\rm{{\rm K}}}\rangle =(\begin{array}{c}{k}_{1}\\ {k}_{2}\\ \vdots \\ {k}_{N}\end{array}),\quad \langle {\rm{{\rm K}}}|=(\begin{array}{cccc}{k}_{1}^{\ast } & {k}_{2}^{\ast } & \cdots  & {k}_{N}^{\ast }\end{array}),$$

where *a*_*N*_ denotes amplitude of the *N*-th mode with resonance frequency *ω*_*N*_, and *k*_*N*_ stands for the coupling between waveguide and cavity.

In Eq. (1), Ω, Γ_*i*_, Γ_*e*_, and Μ matrixes, respectively, denote resonance frequencies, intrinsic loss rate, external loss rate of cavity, and coupling coefficients between resonant modes. They are given as

$$\begin{array}{cc}{\rm{\Omega }}=[\begin{array}{cccc}{\omega }_{11} & {\omega }_{12} & \ldots  & {\omega }_{1N}\\ {\omega }_{21} & {\omega }_{22} & \ldots  & {\omega }_{2N}\\ \vdots  & \vdots  & \ddots  & \vdots \\ {\omega }_{N1} & {\omega }_{N2} & \ldots  & {\omega }_{NN}\end{array}], & {{\rm{\Gamma }}}_{i}=[\begin{array}{cccc}{\gamma }_{i11} & {\gamma }_{i12} & \ldots  & {\gamma }_{i1N}\\ {\gamma }_{i21} & {\gamma }_{i21} & \ldots  & {\gamma }_{i2N}\\ \vdots  & \vdots  & \ddots  & \vdots \\ {\gamma }_{iN1} & {\gamma }_{iN2} & \ldots  & {\gamma }_{iNN}\end{array}],\\ {{\rm{\Gamma }}}_{e}=[\begin{array}{cccc}{\gamma }_{e11} & {\gamma }_{e12} & \ldots  & {\gamma }_{e1N}\\ {\gamma }_{e21} & {\gamma }_{e22} & \ldots  & {\gamma }_{e2N}\\ \vdots  & \vdots  & \ddots  & \vdots \\ {\gamma }_{eN1} & {\gamma }_{eN2} & \ldots  & {\gamma }_{eNN}\end{array}], & {\rm M}=[\begin{array}{cccc}{\mu }_{11} & {\mu }_{12} & \ldots  & {\mu }_{1N}\\ {\mu }_{21} & {\mu }_{22} & \ldots  & {\mu }_{2N}\\ \vdots  & \vdots  & \ddots  & \vdots \\ {\mu }_{N1} & {\mu }_{N2} & \ldots  & {\mu }_{NN}\end{array}].\end{array}$$

In Ω, Γ_*i*_, Γ_*e*_, and Μ matrixes, if *p* ≠ *q*, *ω*_*pq*_ = 0, *γ*_*ipq*_ = 0, *γ*_*epq*_ = 0, and *μ*_*pq*_ = *ω*_*qq*_/(2*Q*_*pq*_); if *p* = *q*, *ω*_*pq*_ = *ω*_*p*_, *γ*_*ipq*_ = *ω*_*pq*_/(2*Q*_*ip*_), *γ*_*epq*_ = *ω*_*pq*_/(2*Q*_*ep*_), and *μ*_*pq*_ = 0. *Q*_*ip*_, *Q*_*ep*_, and *Q*_*pq*_ are cavity quality factors related to intrinsic loss, waveguide coupling loss, and coupling between the *p*th and *q*th modes. Using boundary conditions of *S*_−*in*_ = 0 and Eqs (1–3), one can get the power transmission *T*_*N*_ = |*S*_*+out*_/*S*_*+in*_|^2^ and power reflection *R*_*N*_ = |*S*_−*out*_/*S*_*+in*_|^2^.

When *N* = 1, the power transmission of system is written as

4 $$T={|\frac{j{\omega }_{1}-j\omega +{\gamma }_{i11}}{j{\omega }_{1}-j\omega +{\gamma }_{i11}+{\gamma }_{e11}}|}^{2}.$$

When *N* = 2, the power transmission can be expressed as

5 $$T={|\frac{{t}_{1}{t}_{2}+\frac{{\mu }_{12}{\mu }_{21}}{{\sigma }_{1}{\sigma }_{2}}}{{t}_{1}+{t}_{2}-{t}_{1}{t}_{2}+\frac{{\mu }_{12}{\mu }_{21}}{{\sigma }_{1}{\sigma }_{2}}-\frac{j{\mu }_{12}{k}_{1}{k}_{2}}{{\sigma }_{1}{\sigma }_{2}}-\frac{j{\mu }_{21}{k}_{1}{k}_{2}}{{\sigma }_{1}{\sigma }_{2}}}|}^{2},$$

where *σ*_1_ = (*jω* − *jω*_1_ − *γ*_*i*11_ − *γ*_*e*11_), *σ*_2_ = (*jω* − *jω*_2_ − *γ*_*i*22_ − *γ*_*e*22_), *t*_1_ = 1 + *γ*_*e*11_/*σ*_1_, *t*_2_ = 1 + *γ*_*e*22_/*σ*_2_.

## Plasmonic stub-pair structure

**Figure 2:**
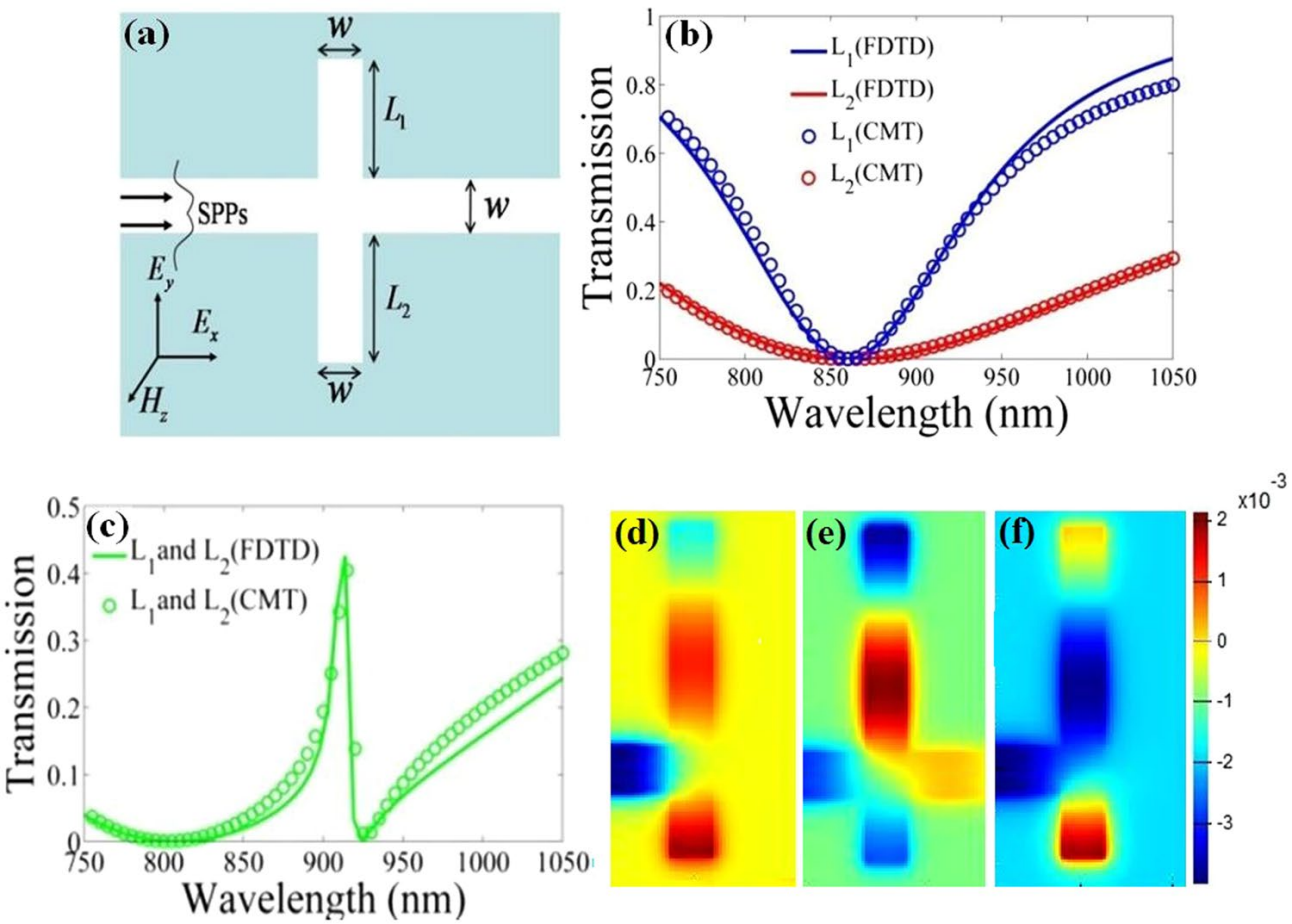
(**a**) Schematic of the plasmonic structure composed of MDM waveguide side-coupled to two stub cavities, with geometrical parameter *w* = 100 nm. (**b**) The blue line (*L*_1_ = 493 nm) and red line (*L*_2_ = 144 nm) correspond to transmission spectra for plasmonic resonator system with single stub. (**c**) Transmission spectra for the plasmonic stub-pair system depicted in Fig. 2(a) with *L*_1_ = 493 nm and *L*_2_ = 144 nm. The solid curves are simulation results and the circles are theoretical fittings. (**d**) Magnetic field distributions (*H*_*z*_) at (**d**) *λ* = 806.7 nm, (**e**) *λ* = 913.7 nm, and (**f**) *λ* = 924.4 nm, respectively, for the plasmonic stub-pair structure.

To verify our theoretical model, the plasmonic stub-pair structure depicted in Fig. 2(a) is taken as an example, which consists of MDM bus waveguide side-coupled to a pair of stub resonators with the width of bus waveguide (*w*), length (*L*_1_ and *L*_2_) and width (*w*) of the stub resonators. The insulator and metal in the structure are, respectively, air and silver. The permittivity of silver, *ε*(*ω*) = 1 − *ω*_*p*_^2^/(*ω* ^2^ + *iωγ*_*p*_), is characterized by the Drude model, with *ω*_*p*_ = 1.38 × 10^16^ rad/s and *γ*_*p*_ = 2.73 × 10^13^ rad/s^34^. The perfect matched layer (PML) boundary conditions are applied to the FDTD simulations, and the spatial steps are set as 5 nm × 5 nm. With incident light polarized parallel to the stubs, surface plasmon mode can be excited and confined in waveguide. In this paper, the widths of bus waveguide and stub cavities are all equal to 100 nm, and transmittance are numerically calculated by the FDTD method.

Figure 2(b) illustrates Lorentzian-like transmission spectra for plasmonic resonator system with single stub. The blue line and red line correspond to *L*_1_ = 493 nm and *L*_2_ = 144 nm, respectively. For these two cases, the resonant wavelengths in Fig. 2(b) are both *λ* = 860.2 nm, and the full width at half maximum (FWHM) of transmission spectra are 155.2 nm and 476.3 nm, respectively. It is well known that the interference between narrowband resonant mode (for the case of single stub system with *L*_1_ = 493 nm) and broadband resonant mode (for the case of single stub system with *L*_2_ = 144 nm) gives rise to Fano resonance^1, 19, 29, 35^. Using the coupled-cavity structure in Fig. 2(a) with *L*_1_ = 493 nm and *L*_2_ = 144 nm, we obtain a sharp asymmetric Fano line shape in transmission spectra, as shown in Fig. 2(c). The transmittances for transmission dips at λ = 806.7 nm and λ = 924.4 nm are 0.00001858 and 0.00263, respectively. In Fig. 2(b) and (c), we present the comparison between the analytical and simulated transmission spectra. For analytical transmission spectra, *ω*_1_ = 2.19 × 10^15^ rad/s, *ω*_2_ = 2.19 × 10^15^ rad/s, *Q*_*i*1_ *= *200, *Q*_*i*2_ = 200, *Q*_*e*1_ = 10, *Q*_*e*2_ = 3, *Q*_12_ = 10. Obviously, the analytical results are in good agreement with the FDTD simulations, which indicates the validity of the theoretical model. Consequently, the theoretical analyses set up a platform to understand the Fano-resonance spectra in coupled cavities system, and may guide design with tunable optical responses.

To better understand the formation mechanisms of Fano line shape, Fig. 2(d–f) show the field distributions (*H*_*z*_) of SPPs at different wavelengths (*λ* = 806.7 nm, *λ* = 913.7 nm, and *λ* = 924.4 nm) in Fig. 2(c). The field distribution in Fig. 2(e) shows that the input light at Fano resonance peak can pass through the waveguide, which arises from the destructive interference between narrowband and broadband resonant modes^1, 19, 20, 35^. Interestingly, Fig. 2(d) and (f) illustrate that the stub-pair can be taken as a composite cavity, in which the resonance modes correspond to transmission dips. Relative to bus waveguide, the mode in phase shown in Fig. 2(d) enhances the coupling between bus waveguide and composite cavity, which corresponds to broadband resonant mode and plays the role of bright mode (quasi-continuum energy state) in conventional Fano system^19^. In Fig. 2(f), the mode out of phase reduces the coupling between bus waveguide and composite cavity, which corresponds to narrowband resonant mode and plays the role of dark mode (quasi-discrete energy state). In other words, the Fano resonance line shape in the plasmonic stub-pair system can also be attributed to the interference between composite cavity modes through the MDM bus waveguide^20, 25, 29, 33^.

## Sensing performance based on Fano resonance

It has been proposed that the asymmetric response line shape with sharp slope in Fano resonance may find applications in label-free, ultra-sensitive, microcavity-based biosensors^21, 36^. The Fano resonance will shift to short-wavelength or long-wavelength direction when the nearby or surrounding medium changes. Here, the resonant wavelength in stub is determined by^37^

6 $$\lambda \approx 4{n}_{eff}L/(2m+1),$$

where *n*_*eff*_, *L*, and *m* (*m* = 0, 1, 2, …) are, respectively, the real part of effective refractive index for SPPs^23, 26^, length, and the order of resonance mode in stub resonator. The resonant wavelength is proportional to *n*_*eff*_ and *L*.

Then, we get

7 $$\frac{d\lambda }{d{n}_{eff}}=4L/(2m+1).$$

For a given detection wavelength, to the refractive index (*n*) of surrounding medium, the derivation of transmission efficiency *T* can be written as

8 $$\frac{dT}{dn}=\frac{dT}{d\lambda }\frac{d\lambda }{d{n}_{eff}}\frac{d{n}_{eff}}{dn}\propto \frac{dT}{d\lambda }.$$

The detection sensitivity of the plasmonic system can be defined as |*dT*/*dn*|. Based on Eqs (7) and (8), for simplicity, the detection sensitivity can also be expressed as^27^

9 $$S\equiv |\frac{dT}{d\lambda }|.$$

**Figure 3:**
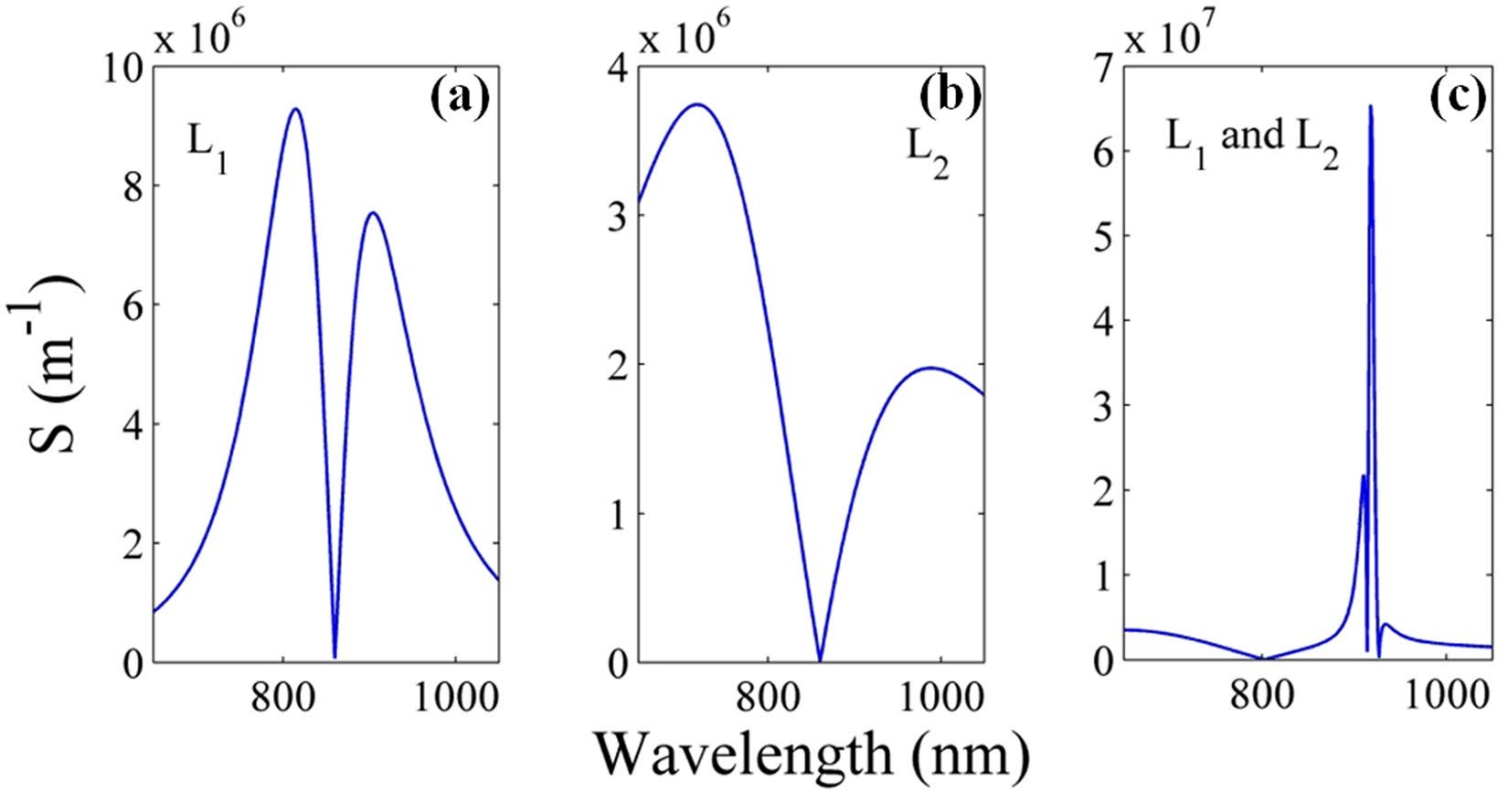
Sensitivity *S* as a function of detection wavelength for three resonator systems: (**a**) single stub *L*_1_, (**b**) single stub *L*_2_, (**c**) stub-pair *L*_1_ and *L*_2_. The system parameters are fixed as *w* = 100 nm, *L*_1_ = 493 nm and *L*_2_ = 144 nm.

According to Eq. (9), we illustrate the sensitivity factor *S* (the slope of transmission spectra versus detection wavelength) as a function of detection wavelength for different plasmonic resonator system in Fig. 3(a–c). Combining Figs 2(b,c) and 3(a–c), we can conclude that the detection sensitivity *S* provides us a way to exhibit spectral asymmetry and investigate the sensitivity of sensor^38, 39^. For the single stub case with *L*_1_ = 493 nm, the detection sensitivity in Fig. 3(a) approaches 9.256 × 10^6^ m^−1^, while the detection sensitivity in Fig. 3(b) reaches 3.742 × 10^6^ m^−1^ for single stub case with *L*_2_ = 144 nm, which indicates that the length of stub resonator offers an efficient way to tune the sensitivity. In Fig. 3(c), for the plasmonic stub-pair system with *L*_1_ = 493 nm and *L*_2_ = 144 nm, the detection sensitivity approaches 6.541 × 10^7^ m^−1^. That is to say, the transmission efficiency will deviate by 0.3271 even when the detection wavelength changes by less than 5 nm. Compared with the single stub in Fig. 3(a) and (b), the stub-pair apparently brings an order of magnitude enhancement in sensitivity.

**Figure 4:**
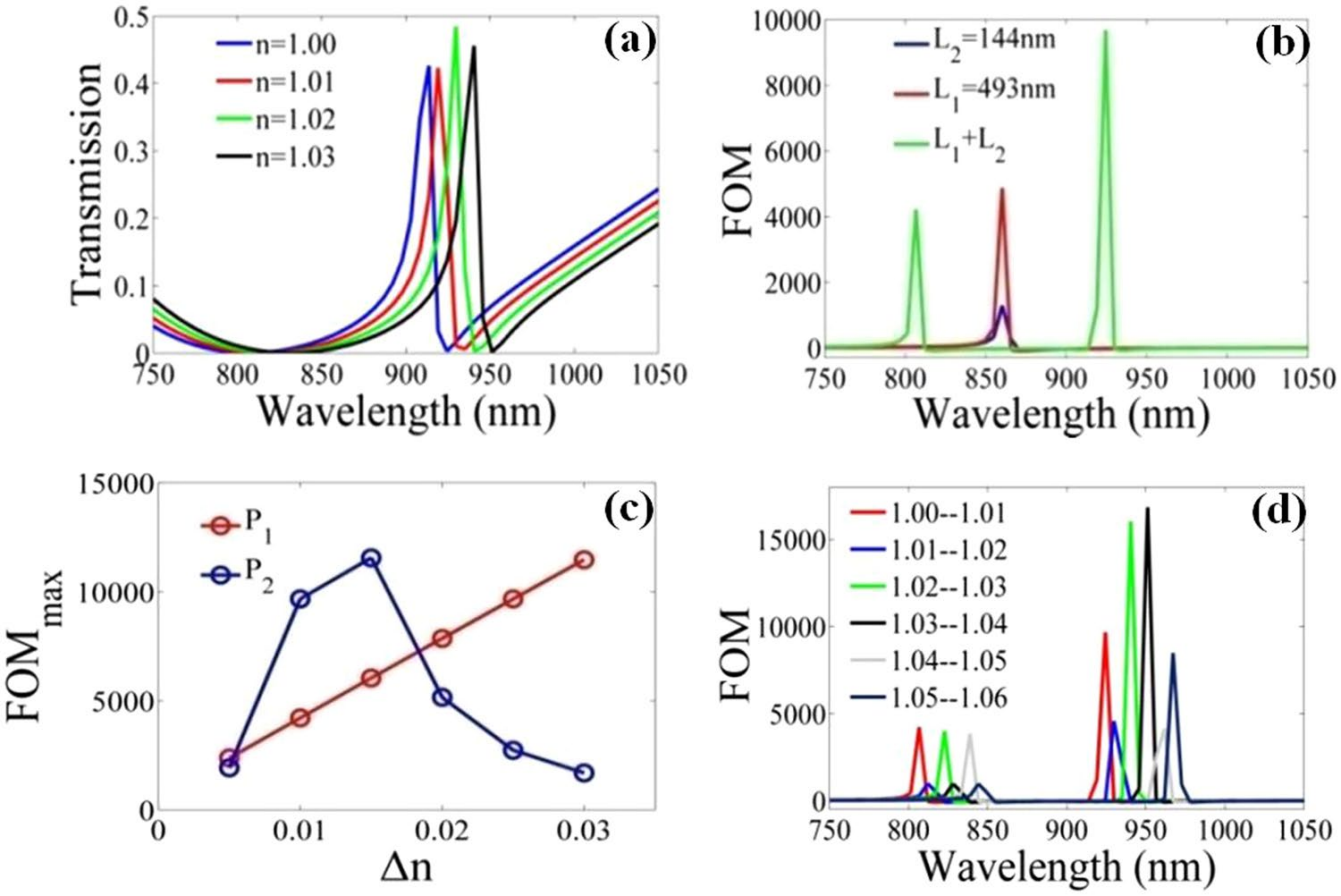
(**a**) Transmission spectra as a function of refractive index of surrounding medium for plasmonic stub-pair structure plotted in Fig. 2(a). (**b**) The curves of FOM for plasmonic stub-pair system (green line) and two corresponding single stub structures (blue line and red line). For the stub-pair structure, (**c**) the maximum of FOM with the change of refractive index Δ*n*, and (**d**) the FOM curves for different ranges of refractive index. The geometrical parameters are fixed as *w* = 100 nm, *L*_1_ = 493 nm and *L*_2_ = 144 nm.

Figure 4(a) shows the transmission spectra versus refractive index of surrounding medium, in which the Fano resonance shifts towards long-wavelength direction with the increase of refractive index, whereas the intensities of Fano resonance peak and resonance dip keep constant. To better characterize the performance of sensing system, the figure of merit (FOM) is one of the key parameters, which can be expressed as FOM = [*T*(*n* + Δ*n*) − *T*(*n*)]*/*[*T*(*n*)Δ*n*] = Δ*T/*(*T*Δ*n*) at fixed wavelength^40–42^. Δ*T* denotes the transmission intensity variation, and Δ*n* stands for the change of surrounding refractive index. *T*(*n* + Δ*n*) and *T*(*n*) are transmission rate in nanoplasmonic structure. As refractive index increases from 1.00 to 1.01, in Fig. 4(b), we plot the FOM for plasmonic stub-pair system and two corresponding single stub structures. For the single stub case with *L*_1_ = 493 nm (*L*_2_ = 144 nm), the maximum FOM is about 4871 (1289); for the stub-pair system, the left and right maximum FOMs (labeled as P_1_ and P_2_, respectively) approach 4218 and 9673, respectively. The results indicate that coupled-cavity offers a method to improve the FOM for refractive index sensing.

In order to investigate the FOM more thoroughly, for the stub-pair structure, Fig. 4(c) shows the maximum of FOM (FOM_max_) versus refractive index *n*. According to the definition of FOM, as refractive index *n* increases from 1.00 to 1.03 with a step of 0.005, *T*(*n*) corresponds to *T*(1.00), and *T*(*n* + Δ*n*) corresponds to *T*(1.005), *T*(1.01), *T*(1.015), *T*(1.02), *T*(1.025) and *T*(1.03), respectively. As shown in Fig. 4(c), when refractive index *n* increases from 1.00 to 1.03, P_1_ increases linearly, but P_2_ increases first and then decreases, which means that FOM_max_ is dependent on the increment of refractive index Δ*n*. In Fig. 4(d), we present the FOM for different ranges of surrounding refractive index, such as 1.01–1.02, 1.02–1.03, 1.03–1.04, 1.04–1.05, and 1.05–1.06. In this case, the change of refractive index Δ*n* equals 0.01, and *T*(*n* + Δ*n*) corresponds to *T*(1.01), *T*(1.02), *T*(1.03), *T*(1.04), *T*(1.05) and *T*(1.06), respectively. It can be obtained that the maximum of P_1_ increases and decreases alternately, while P_2_ approaches 1.6 × 10^4^ in the ranges of 1.02–1.03 and 1.03–1.04. That is to say, the sensing performances are also related to the range of refractive index. These results may lay a basis for fundamental research of ultra-compact plasmonc sensor applications.

**Figure 5:**
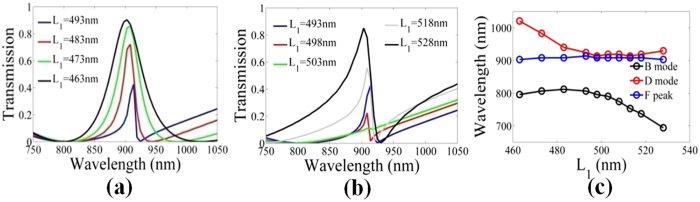
Transmission spectra (**a**) and (**b**) for plasmonic structure in Fig. 2(a) as a function of *L*_1_. (**c**) Wavelengths of bright mode (B mode, black line), dark mode (D mode, red line), and Fano peak (F peak, blue line). The other structure parameters are fixed as *w* = 100 nm, *L*_1_ + *L*_2_ = 637 nm.

It is well known that spectral asymmetry plays a critical role in applications of Fano resonance. Therefore, the control of Fano line shape has been a topic of investigations. Because the Fano resonance arises from the interference between dark mode (narrowband resonant mode) and bright mode (broadband resonant mode), independent control of resonant mode is useful for precise control of Fano resoance in highly integrated optical circuits. In Fig. 5(a) and (b), we present the transmission spectra versus *L*_1_ for the plasmonic stub-pair system. The other constructive parameters are fixed as *w* = 100 nm, *L*_1_ + *L*_2_ = 637 nm. Obviously, the asymmetry of Fano resonance line shape can be adjusted by *L*_1_. To better investigate the evolution of spectral asymmetry versus *L*_1_, we display the wavelengths of bright mode (black line), dark mode (red line), and Fano peak (blue line) as a function of *L*_1_ in Fig. 5(c). As can be clearly seen, when *L*_1_ increases from 463 nm to 528 nm, the Fano peak wavelength is nearly unchanged; when *L*_1_ increases from 463 nm to 493 nm, the wavelength gap between bright mode (dark mode) and Fano peak is nearly constant (becomes smaller); when *L*_1_ increases from 493 nm to 528 nm, the wavelength gap between bright mode (dark mode) and Fano peak becomes larger (is unchanged). As mentioned above, with constant sum of *L*_1_ and *L*_2_ in the plasmomic stub-pair structure, the wavelengths of bright mode and dark mode can be adjusted independently by *L*_1_, which provides an additional degree of freedom to control the slope of Fano resonance spectra and, thus, indicates the tunability of detection sensitivity in the plasmonic nanostructure.

## Conclusion

In summary, we have analytically and numerically investigated tunable and high-sensitivity sensing performances based on Fano resonance in plasmonic coupled cavities system. Based on the CMT, the coupled cavities can be taken as a composite cavity. Using the plasmonic stub-pair structure as an example, the analytical spectral responses are consistent with the FDTD simulations, which confirms our theoretical model, and implies that Fano resonance line shape in plasmonic stub-pair system results from the interference between composite cavity modes through MDM bus waveguide^19, 20, 33^. The detection sensitivity factor in coupled cavities approaches 6.541 × 10^7^ m^−1^, and is an order of magnitude larger than single stub case. Moreover, the effects of structure parameters and surrounding medium play important roles on sensing performances. In particular, the wavelengths of bright mode and dark mode in the plasmomic stub-pair structure can be adjusted independently, which may open up avenues for improving detection sensitivity. These results may be helpful for realizing high-sensitivity sensor in integrated optical circuits.

## Methods

The relative permittivity of silver is described by the Drude model: *ε*(*ω*) = *ε*_*∞*_  − *ω*_*p*_^2^ */(ω* ^2^ + *iωγ*_*p*_), with the dielectric constant at infinite angular frequency ε_*∞*_ = 3.7, the bulk plasma frequency *ω*_*p*_ = 1.38 × 10^16^ rad/s and the electron collision frequency *γ*_*p*_ = *2*.73 × 10^13^ rad/s. The spectral responses of the structure are investigated with the FDTD method using FDTD Solutions. The calculation domain is surrounded by perfectly matched layer (PML) absorbing boundary, and the simulation parameters have been given in our paper.
